# Recurrent wheezing is associated with intestinal protozoan infections in Warao Amerindian children in Venezuela: a cross-sectional survey

**DOI:** 10.1186/1471-2334-14-293

**Published:** 2014-05-29

**Authors:** Marcella MA Overeem, Lilly M Verhagen, Peter WM Hermans, Berenice del Nogal, Adriana Márquez Sánchez, Natacha Martinez Acevedo, Rosalicia Ramirez Murga, Jeroen Roelfsema, Elena Pinelli, Jacobus H de Waard

**Affiliations:** 1 Laboratory of Pediatric Infectious Diseases, Department of Pediatrics, Radboud University Nijmegen Medical Centre, PO Box 9101 (Internal Post 224), 6500 HB Nijmegen, The Netherlands; 2 Laboratorio de Tuberculosis, Instituto de Biomedicina, Universidad Central de Venezuela, al lado del Hospital Vargas, San José, Caracas, Venezuela; 3 Departamento de Pediatría, Hospital de Niños “J.M. de los Rios”, Av. Vollmer, Distrito Capital, Caracas, Venezuela; 4 Escuela José María Vargas, Facultad de Medicina, Universidad Central de Venezuela, Ciudad Universitaria, Distrito Federal, Caracas, Venezuela; 5 Centre for Infectious Disease Control, National Institute for Public Health and the Environment (RIVM), PO Box 1, 3720 BA Bilthoven, The Netherlands

**Keywords:** Atopic eczema, Helminth, Indigenous children, Malnutrition, Protozoa, Recurrent wheezing

## Abstract

**Background:**

While in developed countries the prevalence of allergic diseases is rising, inflammatory diseases are relatively uncommon in rural developing areas. High prevalence rates of helminth and protozoan infections are commonly found in children living in rural settings and several studies suggest an inverse association between helminth infections and allergies. No studies investigating the relationship between parasitic infections and atopic diseases in rural children of developing countries under the age of 2 years have been published so far. We performed a cross-sectional survey to investigate the association of helminth and protozoan infections and malnutrition with recurrent wheezing and atopic eczema in Warao Amerindian children in Venezuela.

**Methods:**

From August to November 2012, 229 children aged 0 to 2 years residing in the Orinoco Delta in Venezuela were enrolled. Data were collected through standardized questionnaires and physical examination, including inspection of the skin and anthropometric measurements. A stool sample was requested from all participants and detection of different parasites was performed using microscopy and real time polymerase chain reaction (PCR).

**Results:**

We observed high prevalence rates of atopic eczema and recurrent wheezing, respectively 19% and 23%. The prevalence of helminth infections was 26% and the prevalence of protozoan infections was 59%. Atopic eczema and recurrent wheezing were more frequently observed in stunted compared with non-stunted children in multivariable analysis (OR 4.3, 95% CI 1.3 – 13.6, p = 0.015 and OR 4.5, 95% CI 0.97 – 21.2, p = 0.055). Furthermore, recurrent wheezing was significantly more often observed in children with protozoan infections than in children without protozoan infections (OR 6.7, 95% CI 1.5 – 30.5).

**Conclusions:**

High prevalence rates of atopic eczema and recurrent wheezing in Warao Amerindian children under 2 years of age were related to stunting and intestinal protozoan infections respectively. Helminth infections were not significantly associated with either atopic eczema or recurrent wheezing.

## Background

The incidence of allergic diseases is rising in industrialized countries and urbanized areas worldwide [1,2]. While genetic factors are important in determining the risk of development of asthma, environmental factors are still likely to be the primary determinants of expression of atopic diseases [3]. Atopic disorders comprise a range of allergic diseases including allergic asthma, anaphylaxis, allergic rhinitis and atopic eczema. Atopic eczema affects approximately 5-20% of children worldwide and mostly children living in urban areas [4,5]. The highest prevalence rates of atopic eczema (more than 15%) are observed in urban Africa, the Baltics, Australia, New Zealand and Northern and Western Europe. The lowest prevalence rates (less than 5%) are observed in China, Eastern Europe, and Central Asia [4]. In several studies a lower prevalence rate of allergic diseases in rural compared to urban children has been observed [5-8]. This difference has been attributed to differences in exposure to aeroallergens and irritant environmental triggers as well as infection with pathogens that can act as allergic disease risk modifiers [9,10].

The so called ‘hygiene hypothesis’ proposes that diminished microbial exposure during early childhood increases susceptibility to allergic diseases [11]. The implementation of hygiene measures as well as the use of antibiotics and vaccines can explain the decreased exposure to different pathogens, particularly in Western countries. Originally, it was proposed that an increased exposure to bacteria and viruses would lead to a strong Th1 response limiting the development of a Th2 immune response, which is typically associated with allergic diseases. However, helminth infections are known to induce a Th2 response and an inverse association between infection with different helminth species and allergic conditions has been reported [12]. It has recently become clear that the interaction between helminth infection and allergic diseases involves regulatory T cells, that dampen both Th1 and Th2 effector responses [13,14].

Although several studies have demonstrated a negative association between intestinal helminth infections and allergic diseases, this association is not consistently observed [2]. In a study including Ethiopian children, there was no significant association between helminth infections and atopic diseases [9], while in pediatric studies from Brazil both positive as well as negative associations between *Ascaris lumbricoides* infection and atopic diseases were observed [15-17]. A systematic review and meta-analysis of epidemiologic studies on the association between intestinal parasite infection and the presence of atopy showed a consistent protective effect of infection with *Ascaris lumbricoides*, *Trichuris trichiura*, hookworm and *Schistosoma* sp. [18].

There are only a few reports assessing the association of protozoan infections with allergic diseases [17,19-21]. *Giardia lamblia* was associated with allergic diseases in children from an urban area of Venezuela [19] while no significant relationship between detection of *G. lamblia* and allergies was observed in urban areas of Brazil [21]. Both studies included children older than 2 years of age. To our knowledge, the relationship between protozoan infections and allergic symptoms has not been investigated in children under 2 years of age.

In rural areas of Venezuela, parasitic infections are commonly observed in children under 16 years of age [22]. Children aged between 12 and 24 months are at a critical stage of growth and development which increases their vulnerability to the detrimental effects of helminth infections. Recent evidence suggests that if interventions to reduce helminth infections and associated malnutrition are not implemented before 24 months of age, future growth and development will be affected [23,24]. Helminthiasis and giardiasis in school-age Venezuelan children were associated with acute and chronic nutritional status respectively [25]. The vicious cycle of malnutrion and parasitic infections is associated with immune modulation. A disturbed immune balance may play a role in the development of immune-mediated diseases like atopy.

We performed a cross-sectional survey to study the association of helminth infections, protozoan infections and malnutrition with atopic eczema and recurrent wheezing in Warao Amerindian children aged 0 to 2 years.

## Methods

### Study population

The Delta Amacuro or Orinoco Delta comprises 4 municipalities. This cross-sectional survey was undertaken in 11 geographically spread Warao villages, located in the largest of the 4 municipalities, Antonio Diaz, which has almost 19,000 inhabitants. Warao Amerindian children aged between 0 and 2 years were included. With a population of 155,000, the Warao people are the second most important Native American group in Venezuela. They live in about 300 geographically isolated villages that are spread throughout this area, where they receive little medical attention and live under precarious sanitary conditions, experiencing a high incidence of infectious diseases.

### Data collection

This study was carried out from August 1, 2012 to November 7, 2012. First, all children attending health clinics in the 11 villages were recruited. Subsequently, door-to-door visits were conducted in each of the 11 communities. The investigators stayed in every community for approximately three to seven days. If an individual was not home during the door-to-door visits, return visits were made to include all age-eligible children living in the communities at the time of survey. After written informed consent and the collection of demographic information, face-to-face interviews using a standardized questionnaire were conducted with parents or guardians of the children. The history included questions concerning age, sex and habitation of the child. A physical examination including anthropometric measurements of the child and inspection of the skin was performed and documented on a standardized data collection sheet. Children were measured for height and weight without shoes and in light indoor clothing using a tape measure with a precision to 0.1 cm. Weight was measured using an electronic digital scale, with a maximum capacity of 150 kg and a precision to 0.1 kg. A stool sample was requested from all participants. Stool samples were preserved in sodium acetate-acetic-formalin (SAF) preservative [26] and stored at 4°C until microscopical examination by experienced laboratory technicians for the presence of helminths and intestinal protozoa. An aliquot of the unpreserved sample was mixed with ethanol 96%. Fecal samples in ethanol were stored at -70°C until DNA isolation with the High Pure PCR Template Preparation Kit (Roche, Almere, The Netherlands). Real-Time PCR of fecal samples was performed on the Roche LightCycler 480 system using the Roche FastStart Kit. Detection of the protozoa *Dientamoeba fragilis*, *Giardia lamblia* and *Cryptosporidium* sp. was done in one multiplex PCR with Phocine Herpes Virus (PhHV) as an internal control using primers and probes described by Verweij et al. [27,28] PCR on the nematodes *Ascaris lumbricoides*, *Strongyloides stercoralis*, *Ancylostoma duodenale* and *Necator americanus* was also performed in a multiplex PCR with primers and probes described by Basuni et al. [29]. The primers and probes sequences are listed in Table 1, including the amount that was used in a single reaction. All PCRs were carried out in 25 μl reactions in the presence of 4 μg BSA fraction V (Sigma-Aldrich, Zwijndrecht, The Netherlands). After an initial activation step of 10 minutes at 95°C the reaction consisted of 45 cycles at 95°C for 10 seconds, 58°C for 20 seconds and 72°C for 20 seconds.

**Table 1:** Multiplex PCR on protozoa and nematodes

**Name**	**Sequence (label & quencher)**	**Amount**	**Target organism**
Multiplex PCR on protozoa			
Crypto-tp	Tex Red-ccaatcacagaatcatcagaatcgactggtatc-BHQ2	5 pmol	*Cryptosporidium* sp.
Crypto CrF	cgcttctctagccttttcatga	15 pmol	
Crypto CrR	cttcacgtgtgtttgccaat	15 pmol	
Giardia-tp	Fam-cccgcggcggtccctgctag-BHQ1	1 pmol	*G. lamblia*
Giardia 80 F	gacggctcaggacaacggtt	5 pmol	
Giardia 127R	ttgccagcggtgtccg	5 pmol	
Df-172	Vic-caattctagccgcttat-BHQ1	5 pmol	*D. fragilis*
Df 124-F	caacggatgtcttggctcttta	14 pmol	
Df 221-R	tgcattcaaagatcgaacttatcac	14 pmol	
PhHV TP	Cy5-tttttatgtgtccgccaccatctggatc-BHQ2	7.5 pmol	Phocine Herpes virus
PhHV-F	gggcgaatcacagattgaatc	10 pmol	
PhHV-R	gcggttccaaacgtaccaa	10 pmol	
Multiplex PCR on nematodes			
Ad155MGB-TP	Fam-atcgtttaccgactttag-BHQ1MGB	2.5 pmol	*A. duodenale*
Ad125F	gaatgacagcaaactcgttgttg	5 pmol	
Ad195R	atactagccactgccgaaacgt	5 pmol	
Na-TP	Tex Red-gtgttcagcaattcccgtttaagtgaag-BHQ2	1.25 pmol	*N. americanus*
Na58F	ctgtttgtcgaacggtacttgc	5 pmol	
Na158R	ataacagcgtgcacatgttgc	5 pmol	
Alum124T-TP	Vic-ttggcggacaattgcatgcgat-BHQ1	1.25 pmol	*Ascaris* sp.
Alum96F	gtaatagcagtcggcggtttctt	2 pmol	
Alum183R	gcccaacatgccacctattc	2 pmol	
Stro TP	ATTO425-acacaccggccgtcgctgc-BHQ1	1.25 pmol	*S. stercoralis*
Stro18s-1530F	gaattccaagtaaacgtaagtcattagc	2.5 pmol	
Stro18s-1630R	tgcctctggatattgctcagttc	2.5 pmol	

### Definitions of atopic eczema, recurrent wheezing and malnutrition

Eczema was investigated by a questionnaire and physical examination using standardized instruments based on the United Kingdom Working Party (UKWP) criteria/Nottingham protocol [30]. Cases of flexural dermatitis were more specifically assessed for severity using the SCORAD protocol [31]. Eczema was defined by at least one presentation during the first 2 years of life of an itchy skin condition plus 3 or more of the following: 1) history of involvement of flexural sites including the cheeks; 2) history of atopic disease in a first degree relative; 3) history of generally dry skin in last year and 4) visible flexural dermatitis or dermatitis affecting the cheeks or forehead and outer limbs (Table 2) [30]. These strict criteria were used since in these communities other causes of “itchy skin” are common (e.g. scabies, insect bites or helminth induced rashes); for the same reason the diagnosis of atopic eczema always included positive findings upon medical examination of the child. Wheezing was defined as recurrent wheezing when the child suffered from two or more wheezing episodes in the past 12 months. Recurrent wheezing was only assessed in children aged 12 months and above (n=123). The anthropometric measurements were transformed into weight-for-age (WAZ), height-for-age (HAZ), and weight-for-height (WHZ) *Z* scores based on WHO standard reference populations using WHO Anthro software [32,33]. Malnutrition was defined as underweight (WAZ < -2 SD), stunting (HAZ < -2 SD), and wasting (WHZ < -2 SD) [32].

**Table 2:** Questionnaire used for defining atopic eczema

**Question:**	**Yes, n%**	**No, n%**
1a. In the last year, has your child had an itchy skin condition – by itchy we mean scratching or rubbing the skin?	90 (39)	139 (61)
1b. Has your child has an itchy skin condition in the last week?	58 (65)	30 (35)
2. Has the skin condition ever affected the skin creases in the past - by skin creases we mean one of the following condition you see below:	65 (72)	25 (28)
Fronts of elbows	36 (40)	54 (60)
Behind the knees	29 (32)	61 (68)
Fronts of ankles	32 (36)	58 (64)
Around the neck	41 (46)	49 (54)
Around the eyes	19 (21)	71 (79)
On the cheeks	21 (23)	69 (77)
On both forearms	31 (35)	59 (65)
On both legs	33 (37)	57 (63)
3. Does anyone in your child’s immediate family (mother, father, brother or sister) suffer from eczema, hay fever or asthma?	84 (37)	144 (63)
4. In the last year has your child suffered from a dry skin in general?	42 (18)	186 (82)
Physical examination:	Flexural dermatitis	Dermatitis cheeks/forehead and outer limbs
5. Is there visible flexural dermatitis or dermatitis affecting cheeks/forehead and outer limbs?	66 (29)	81 (35)

Eczema was defined by at least one presentation during the first 2 years of life of an itchy skin condition (question 1a) plus 3 or more of the following: 1) history of involvement of flexural sites including the cheeks (question 2); 2) history of atopic disease in a first degree relative (question 3); 3) history of generally dry skin in last year (question 4) and 4) visible flexural dermatitis or dermatitis affecting the cheeks or forehead and outer limbs. (question 5).

### Statistical analysis

Categorical variables were analyzed using univariate analysis. To determine which characteristics were associated with atopic eczema and recurrent wheezing, multivariable logistic regression models were built. The variables of interest (i.e. atopic eczema, recurrent wheezing, malnutrition, protozoan infections and helminth infections) were included in the multivariable model irrespective of their p-values in the univariate analysis. Age and gender were included in the multivariable model if p <0.20 in univariate analyses. Statistical analyses were performed using the SPSS program for Windows version 20.0 (SPSS Inc, Chicago, IL).

### Ethical considerations

The nature and objectives of the study were explained to the parents of children in Spanish and/or in their native language. This study was a pilot study within a larger study on response to pneumococcal vaccination which was approved by the ethical committee of the Instituto de Biomedicina, Caracas 1010A, issued on the 22th of March 2011 in Caracas, Venezuela. Furthermore, the study was approved by the Servicio de Atención y Orientación Indígena (S.A.O.I) on the 2th of December 2010 in Tucupita, Venezuela. Children were enrolled if their parents or primary caregivers provided written informed consent. If parents or guardians did not speak Spanish, the questions were translated into the native Warao language by local translators. The aims of the research and the definitions and explanations of the terms used were discussed with community elders, community health workers and local translators prior to the inclusion of patients. Preliminary testing of the questionnaires was then carried out by the local translators together with the survey staff. After these test interviews, the questionnaire was slightly modified to adapt it to the local terminology. If parents or primary caregivers were illiterate, consent forms were read to them in Spanish and/or in their native language and they were signed by means of a thumb print. Anti-helminthic and anti-protozoal treatment based on the feces microscopy results was provided free of charge by the study team.

## Results

From August to November 2012, 229 children 0 to 24 months of age were included. All parents/guardians were willing to answer questions and cooperate with physical examination of the child. Fecal samples were collected from 100 children (44%). Children from whom stool samples were obtained were more likely to suffer from stunting than children from whom stool samples were not obtained (56% vs. 40%, p = 0.013). Characteristics of the study population are shown in Table 3.

**Table 3:** Characteristics of the study population (n = 229)

Age (months), mean (SD),	13 (6)
Male	115 (50)
Female	114 (50)
Community, n%	
Araguabisi	16 (7)
Araguaimujo	27 (12)
Arature	21 (9)
Bonoina	24 (11)
Guayaboroina	9 (4)
Guayo	26 (11)
Ibaruma	20 (9)
Jobure de Curiapo	31 (14)
Merejina	14 (6)
Nabasanuka	26 (11)
Winikina	15 (7)
Malnourished: stunting (HAZ < -2 SD), n%	107 (47)
Malnourished: underweight (WAZ < -2 SD), n%	41 (18)
Malnourished: wasting (WHZ < -2 SD), n%	15 (7)
Atopic eczema, n%	43 (19)
Recurrent wheezing in children ≥ 12 months of age, n%	28 (23)
Helminth infection, n%	26 (26)
*Ascaris lumbricoides*	15 (15)
*Trichuris trichiura*	4 (4)
*Strongyloides stercoralis*	11 (11)
*Necator americanus*	2 (2)
*Ancylostoma duodenale*	1 (1)
Protozoan infection, n%	59 (59)
*Giardia lamblia*	47 (47)
*Dientamoebe fragilis*	4 (4)
*Cryptosporidium parvum*	15 (15)
*Blastocystis hominis*	5 (5)
*Iodamoeba sp*	1 (1)
*Entamoeba dispar*	1 (1)
*Chilomastix mesnili*	1 (1)

The prevalence of atopic eczema in the study population was 19% (n = 43) and recurrent wheezing was observed in 23% (n = 28). A borderline significant trend towards more atopic eczema in children suffering from recurrent wheezing was observed (32% vs. 16%, p = 0.055). Infection with an intestinal parasite was present in 70% (n = 70) of the children that handed in fecal samples. In 26 children (26%), a helminth infection was detected while 59 children (59%) suffered from a protozoan infection. *G. lamblia* was the most frequently detected pathogen, infecting 47 of the included children (47%). Characteristics of included children and the association between parasitic infections and atopic eczema and recurrent wheezing are presented in Tables 4 and 5. There were no significant differences regarding age or sex between the children with or without atopic eczema. The prevalence of atopic eczema was significantly higher in stunted compared with non-stunted children in multivariable analysis (OR 4.3, 95% CI 1.3 – 13.6, p = 0.015).

**Table 4:** Characteristics of Warao Amerindian children 0 to 2 years of age, malnutrition and parasitic infections compared with atopic eczema*

**Characteristics**	**Eczema**	**No eczema**	**Univariate analysis**		**Multivariable analysis**
	**(n=23)**	**(n=77)**	**p-value**	**OR (95% CI)**	**OR (95% CI)**
Age (months), mean (SD)	14 (6)	13 (6)	0.38	1.0 (0.96-1.1)	#
Sex, n (%)			0.62		
Male	13 (57)	39 (51)		1	#
Female	10 (43)	38 (49)		0.79 (0.31-2.0)	
Stunting vs. non-stunting (HAZ < -2 SD), n (%)	18 (78)	38 (49)	0.018	3.7 (1.2-11.0)	4.3 (1.3-13.6)
Underweight vs. not underweight (WAZ < -2 SD), n (%)	5 (22)	17 (22)	0.97	0.98 (0.32-3.0)	0.52 (0.15-1.8)
Wasting vs. non-wasting (WHZ < -2 SD), n (%)	0 (0)	6 (8)	#	#	#
Parasitic infections, n (%)					
Helminth infection present vs. absent	6 (26)	20 (26)	0.99	1.0 (0.35-2.9)	0.98 (0.32-3.0)
Protozoan infection present vs. absent	11 (48)	48 (62)	0.22	0.55 (0.22-1.4)	0.69 (0.26-1.8)

*These analyses were performed on children from who we collected stool samples (n=100).

**Table 5:** Characteristics of Warao Amerindian children 0 to 2 years of age, malnutrition and parasitic infections compared with recurrent wheezing*

**Characteristics**	**Recurrent wheezing**	**No recurrent wheezing**	**Univariate analysis**		**Multivariable analysis**
	**(n=16)**	**(n=45)**	**p-value**	**OR (95% CI)**	**OR (95% CI)**
Age (months), mean (SD)	18 (3)	17 (4)	0.57	1.0 (0.90-1.2)	#
Sex, n (%)			0.10		
Male	13 (81)	26 (58)		1	1
Female	3 (19)	19 (42)		0.32 (0.079-1.3)	0.28 (0.061-1.3)
Stunting vs. non-stunting (HAZ < -2 SD), n (%)	12 (75)	25 (56)	0.18	2.4 (0.67-8.6)	4.5 (0.97-21.2)
Underweight vs. not underweight (WAZ < -2 SD), n (%)	4 (25)	12 (27)	0.90	0.92 (0.25-3.4)	0.78 (0.15-4.1)
Wasting vs. non-wasting (WHZ < -2 SD), n (%)	0 (0)	4 (9)	#	#	#
Parasitic infections, n (%)					
Helminth infection present vs. absent	10 (63)	27 (60)	0.86	0.90 (0.28-2.9)	0.77 (0.20-3.0)
Protozoan infection present vs. absent	13 (81)	21 (47)	0.024	5.0 (1.2-19.8)	6.7 (1.5-30.5)

*These analyses were performed on children from who we collected stool samples (n=61).

The prevalence of recurrent wheezing in the last 12 months was 21% (n=48) and for children aged 12 months and above, it was 23% (n=28). There were no significant differences regarding age or sex between the children with or without recurrent wheezing. Stunting was more often observed in children with recurrent wheezing compared with children without recurrent wheezing, but this difference was not statistically significant in multivariable analysis (OR 4.5, 95% CI 0.97 - 21.2, p = 0.055). Children with recurrent wheezing were significantly more often infected with a protozoan infection compared with children that did not suffer from recurrent wheezing in multivariable analysis (OR 6.7, 95% CI 1.5 – 30.5).

## Discussion

This cross-sectional survey showed that Warao Amerindian children in Venezuela aged 0 to 2 years suffer from a high prevalence of atopic eczema and recurrent wheezing. Recurrent wheezing prevalence rates were significantly positively associated with protozoan infections while rates of atopic eczema were significantly higher in stunted children. Helminth infections were not significantly associated with either atopic eczema or recurrent wheezing.

The prevalence of recurrent wheezing in our study population was 23%. Similar prevalence rates of recurrent wheezing in children aged 0 to 2 years were observed in urban areas of Brazil and Norway [34,35], although study definitions regarding the minimal number of wheezing episodes (respectively three in the first year of life and two in the first two years of life) were not identical to our study. In a rural area of Colombia, the prevalence of recurrent wheezing (≥3 episodes) in children aged 24 months was 14% [36]. Definitions of recurrent wheezing in young children used in different studies vary widely. Our definition of recurrent wheezing has been used previously in preschool children [37,38]. In urban areas with hospitals keeping patient records, questionnaires for recurrent wheezing in infants can be validated by comparing the parental answers to questionnaires with diagnoses made by pediatric respiratory specialists and this has led to the use of such questionnaires in urban health centres in Latin America [39,40]. The diagnostic value of these questionnaires in rural indigenous populations remains to be seen.

Almost one fifth of the children included in this survey were diagnosed with atopic eczema (19%). In Shanghai in children 3 to 6 years of age an overall atopic eczema prevalence of 8% was observed, ranging from 5% in rural areas to 13% in urban areas [5]. In Europe, the overall prevalence of atopic dermatitis in 1133 children living in five European countries in farming and non-farming rural areas was 16% (range 8%-30%) [41].

We observed a significantly higher prevalence of atopic eczema in children suffering from stunting compared with children who were not stunted and a trend towards more stunting in children with recurrent wheezing compared to children without recurrent wheezing. Stunting indicates chronic malnutrition. In children with malnutrition, cell-mediated (T-cell) immunity, IgA levels in secretions, complement levels, and phagocytosis are all diminished [42]. Whether stunted children with an altered immune response have a higher risk of atopic reactions than non-stunted children warrants further investigation. In the group of children with wasting, indicating acute malnutrition, we did not record cases of atopic eczema and only two cases of recurrent wheezing. As this acute presentation of malnutrition develops in a short period of time, it is possible that malnutrition-related alterations in the immune response that might be associated with the development of atopic eczema have not yet developed in children with an acute form of malnutrition.

The overall gastrointestinal parasitic infection prevalence in children 0 to 2 years of age in our study was 70%. The prevalence of helminth infections was 26%. Similar helminth infection prevalence rates in children under 2 years of age were observed in rural areas in other South American countries [24,43,44]. The prevalence of gastrointestinal protozoan infections in our study population was 59% with *G. lamblia* as the most prevalent protozoan infection, infecting 47% of the children. The prevalence of *G. lamblia* infections we observed is higher than prevalence rates in an earlier study performed in the Orinoco Delta between 2010 and 2011. This study included 141 children aged 1 to 15 years and recorded a protozoan infection prevalence of 65% and a *G. lamblia* infection prevalence of 37% [22]. In pre-school children, a higher prevalence of *G. lamblia* infections in the rainy season compared with the dry season has been observed [45]. Our study was performed in the rainy season while the earlier study was performed during a whole year, which can explain the higher prevalence of *G. lamblia* in our study compared to the earlier study*.* In other South American countries the prevalence of *G. lamblia* in children aged 0 to 2 years is approximately 20% [43,44]. However, these studies detected *G. lamblia* infections only microscopically. We detected *G. lamblia* by both microscopy and real time PCR, a technique with a very high sensitivity for protozoan infections [28].

The presence of protozoan infections was significantly associated with a high prevalence of recurrent wheezing in our study. Two earlier studies performed in Venezuela in 1993 and 1998 showed similar positive associations between *G. lamblia* and allergic diseases [19,20]. A recent Brazilian study showed no association between *G. lamblia* and symptoms of asthma or skin allergy [21]. However, all of these studies included children older than 2 years of age. Helminth infections were not associated with either recurrent wheezing or atopic eczema in our study while an inverse association between infection with different helminth species and allergic conditions has been reported in other studies [16-18] However, the children included in these studies were also older than 2 years. The immune system of infants and young children shows great plasticity and is more amenable to modulation than the immune system of older children and adults [46]. Age is therefore likely to be a major determinant of the interaction between intestinal parasitic infections and the host immune respons and more studies including children younger than 2 years of age are warranted.

The interpretation of a diagnosis of recurrent wheezing in children younger than 2 years of age is questionable. Wheezy lower respiratory tract illnesses are common in early childhood and are often associated with viral infections, particularly with respiratory syncytial virus (RSV) [47,48]. Recurrent wheezing in our study children could thus be the mere result of repeated viral infections rather than a marker for bronchial allergy. However, even if recurrent wheezing was caused by viral respiratory tract infections, wheezing episodes could still be related to an allergic predisposition of the child. Prospective studies show that infants with repeated episodes of viral bronchiolitis often develop allergic asthma during childhood [49-51] and it has been suggested that a predisposition for bronchial obstructive disease underlies both recurrent wheezing in young children and asthma development during childhood [52]. In a recently published large prospective birth cohort study, two-third of the children with recurrent wheezing episodes by 2 years of age was diagnosed with asthma or bronchial hyper responsiveness or used asthma medication at 16 years of age [35]. Atopy as measured by skin prick tests and atopic dermatitis in infancy has also been identified as a predictor for the subsequent development of asthma [51,53]. Another study among schoolchildren living in a poor rural region of tropical Latin America, showed a predominance of non-atopic compared with atopic wheeze. However the subjects of this study were between 6 and 16 years old and thus not comparable to our study population [54]. The positive association between atopic eczema and recurrent wheezing that we observed indicates that recurrent wheezing in our studied children could be a marker for susceptibility for atopic asthma. However, follow-up studies in this population at an older age are required to establish a possible association of recurrent wheezing with subsequent asthma development. Furthermore, it is recommendable to include other measures of atopy such as skin prick tests in future follow-up studies.

In this survey a stool sample was collected in 100 of the 229 subjects (44%). The prevalence of stunting was higher in children from whom stool samples were obtained than in children without a stool sample. A possible explanation for this finding is that parents of malnourished children were more likely to collect stool samples because of concerns about the state of health of their children.

The diagnosis of atopic eczema was based on the Nottingham- and SCORAD-protocol [30,31]. Both instruments are designed for diagnosing clinical atopic dermatitis. Additional information about the skin condition and familiar predisposition was obtained in order to distinguish between atopic dermatitis and other skin conditions such as scabies and viral or antibiotic rash. However, we cannot exclude the possibility that the presence of other skin conditions has led to false-positive observations and overreporting of the prevalence rate of atopic eczema. We observed that atopic eczema in children was often not identified as a problem by their parents. Dermatitis and rash were attributed to sweating and often no treatment was given, even if a topical treatment was provided by a local nurse. Severe cases of dermatitis were recognized as aberrant and a reason to consult a doctor. Scratching, which can easily lead to secondary infections in this setting, was generally not considered to be a problem. As atopic eczema in young children can lead to significant health problems [55], qualitative studies are needed to determine whether parents have disease awareness of atopic eczema. Possibly, there is a need for health and educational services on this topic.

In addition to the already mentioned limitations, eg the interpretation of the diagnosis of recurrent wheezing and the lack of blood samples or skin-prick tests, there are several other limitations to our study, some of which are related to the challenging logistics of conducting an epidemiological study in an area with a poor infrastructure characterized by low literacy and poor access to health care. Although we performed a multivariable analysis taking into account possible confounders such as age and sex, unmeasured factors may have caused residual confounding. Male gender, low birth weight (<2,500 g), low gestational age (<37 weeks), breastfeeding for less than 6 months, congenital heart disease, family history of atopy, asthma, smoking exposure and stove warming, have been identified as significant risk factors for recurrent wheezing while the presence of pets at home seems to be a protective factor [56]. It is difficult to obtain information on birth weight and gestational age in the Warao population, because documentation on new-borns is not collected consistently. Most women deliver their children at home and do not visit a hospital. There are no records available on gestational age because women generally do not know their exact pregnancy duration. As virtually all Warao children are breastfed until at least 12 months of age, it is not likely that the lack of information on duration of breastfeeding has influenced our study results. The prevalence of congenital heart disease is unknown. Also, the lack of information on smoking exposure and the presence of pets in the household may have caused residual confounding.

Additionally, although included villages were chosen to be geographically spread throughout the Antionio Díaz municipality, no randomization procedure was performed for the selection of villages. If reported wheezing episodes were related to viral infections, substantial variation between communities in the prevalence of recurrent wheezing could be associated with the rapid spread of viral infections within communities. Because most Warao people live with several families together in one house, viral infections spread rapidly, leading to a rise in overall prevalence when a few community members become infected. Finally, the use of a questionnaire may have caused recall bias. However, a questionnaire is the only available instrument in the Warao population because hospital data on atopic diseases are scarce due to the lack of personal medical records.

## Conclusions

Although it is generally assumed that in rural areas of developing countries allergic diseases are relatively uncommon compared to industrialized areas of developed and developing countries, a high prevalence of atopic eczema and recurrent wheezing in Warao children 0 to 2 years of age was observed. Chronic malnutrition was significantly associated with a high prevalence of atopic eczema in multivariable analysis. Protozoan infections were significantly associated with the presence of recurrent wheezing in multivariable analysis. No significant associations between helminth infections and atopic eczema or recurrent wheezing were observed. The immune regulatory mechanisms involved in the association between intestinal protozoan infections and allergic manifestations require further investigation. An integrated approach including improvement of the sanitary conditions, boosting agricultural productivity and creating awareness is needed to improve the health status and eliminate intestinal parasitic infections and immunopathologies in young Warao Amerindian children.

## Competing interests

The authors declare that they have no competing interests.

## Authors’ contributions

MO participated in the design of the study, the collection of data, the statistical analysis, the interpretation of data and drafting the manuscript. LV participated in the design of the study, the collection of data, the statistical analysis, the interpretation of data, drafting the manuscript and revising it critically. PH participated in the design of the study and revised the manuscript critically for important intellectual content. BN advised on patient recruitment and revised the manuscript critically for important intellectual content. AM participated in the collection of data and helped to draft the manuscript. NA participated in the collection of data and helped to draft the manuscript. RM carried out fecal microscopical analysis. JR participated in the data analysis and helped with the PCR of feces. EP advised on data analysis and revised the manuscript critically for important intellectual content. JW participated in the design of the study, coordinated the field work, and revised the manuscript critically for important intellectual content. All authors read and approved the final manuscript.

## Pre-publication history

The pre-publication history for this paper can be accessed here:

http://www.biomedcentral.com/1471-2334/14/293/prepub
